# In guidelines physicians trust? Physician perspective on adherence to medical guidelines for type 2 diabetes mellitus

**DOI:** 10.1016/j.heliyon.2020.e04803

**Published:** 2020-08-31

**Authors:** Sophie Brenner, Willi Oberaigner, Harald Stummer

**Affiliations:** UMIT - Private University for Health Sciences, Medical Informatics and Technology, Hall in Tirol, Austria

**Keywords:** Endocrinology, Evidence-based medicine, Health informatics, Health profession, Health promotion, Health technology, Diabetes mellitus, Type 2, Guideline adherence, Physician-patient relationship, Information systems

## Abstract

**Aims:**

Adherence to treatment guidelines and treatment success are low in Type 2 diabetes mellitus (T2DM). This study aims to capture the physician perspective on T2DM guideline adherence and identify levers for increasing adherence.

**Methods:**

A survey among German physicians captured the perceived value of 4 areas in the national treatment guideline (NVL), 13 possible barriers, and 9 possible enablers for guideline adherence. Perceived value was assessed by ranking 4 NVL areas by implementation difficulty and impact on treatment success. Barriers and enablers were assessed by rating their influence on guideline deviation and adherence. The consistency of results across subgroups was assessed using Fisher's exact test.

**Results:**

Responses from 46 physicians showed a strong consensus about the value of each NVL area. Physicians perceived patient inability and demotivation to be the strongest adherence barriers (93%, 78%). All queried enablers were approved by ≥ 50% of participants. Physicians considered cross-provider collaboration and electronic therapy decision support as strongest enablers (85%, 80%). Consistency was high between subgroups.

**Conclusion:**

This study suggests that physicians consider patient-related factors to be stronger barriers for guideline adherence than physician-related factors. Finding opportunities to increase physician buy-in is important for better guideline adherence. In this study, physicians voiced appreciation for adherence enablers based on digital solutions to support the care process and to reduce the complexity of therapy decisions.

## 1 Introduction

Treatment success in diabetes is not satisfactory. Glycemic control, i.e. achieving target levels of glycated hemoglobin (HbA1c), is low [1, 2]. Internationally, only about 40–60% of patients are achieving glycemic control [3]. Yet, previous studies have established that even mild hyperglycemia, over a longer period, leads to diabetes complications. Also, early treatment of hyperglycemia has favorable, long term effects on complications [3, 4].

To address hyperglycemia and diabetes complications, medical guidelines have been issued. They aim to embed the latest scientific knowledge and best practices in daily medical practice, to improve treatment success. Guidelines are formally established in most countries but implementation success and monitoring of guideline adherence are lacking [5].

Adherence rates to T2DM guidelines are low, typically at around 40–60%. Often, guideline adherence is measured by assessing which share of participants has received a lab test or examination (e.g. annual HbA1c screening, foot examination). In some cases, adherence is measured longitudinally by assessing whether a treatment algorithm was followed or by evaluating whether certain structural requirements of the guideline were met [6, 7, 8, 9].

Achieving guideline-adherent disease management depends on patient factors, health care system factors, and physician factors. Patient factors include e.g. health literacy, lifestyle, or awareness of the disease. Health care system factors include e.g. availability of disease registry, collaboration among the interdisciplinary care team, or visit planning and follow-up. Physician factors include e.g. time for patient care, the ability to set clear treatment goals, and reactive vs. proactive approach to care [10, 11, 12]. There is much less information available about physician factors leading to non-adherence than about patient and system factors. Physicians have a significant influence on achieving guideline adherent care, and thus, treatment success [3].

In Germany, 7.5 M adults (20–79 years) are supposed to have diabetes, 90% of them T2DM. An estimated 34% of these cases are undiagnosed. 11% of annual deaths are caused by diabetes, which is more than all infectious diseases combined [13].

Since 2002, Germany has a national guideline program for T2DM which consists of 6 guidelines. The national T2DM treatment guideline (NVL) was last revised in 2014. A consolidated update of the guideline program is currently under review and expected to be published by the end of 2020.

Diabetes care in Germany is provided by general practitioners (GP), diabetology specialists, and hospitals. 80–90% of patients with diabetes are monitored and treated by their GPs. The remaining patients are in the temporary or permanent care of one of 1,100 specialist practices. There, trained diabetologists manage uncontrolled episodes of diabetes or other more challenging cases. Physicians are supported by trained diabetes staff including diabetes consultants, diabetes assistants, and diabetes nurses. Emergencies, glycemic escalations, and severe complications require patients to be admitted to hospitals with specific qualifications to treat diabetes [14].

Since 2003, Germany has a disease management program (DMP) for outpatient T2DM care, which aims to improve the health of T2DM patients. The DMP coordinates and evaluates evidence-based T2DM care across providers. In 2019, 4.3m patients participated in the DMP [15]. A GP in Germany spends less than 8 minutes with each patient. He also spends 7 hours per week with non-face-to-face medical tasks like documentation, medical opinion statements, and case conferences [16, 17]. GPs treat a broad spectrum of conditions with only 14% of patients having a T2DM diagnosis [18].

At the same time, optimal care becomes more complex. Physicians are faced with a large number of treatment options to choose from: In the past 10 years, the European Medicines Agency alone has approved 35 new medicines for diabetes treatment [19]. New research results lead to more differentiated treatment recommendations: The American Diabetes Association (ADA) and European Association for the Study of Diabetes (EASD) now recommend choosing the treatment approach based on the primary diabetes comorbidity [20].

In daily practice, most physicians work with administrative IT systems. These systems serve billing purposes, can document the care process, and have some reporting capability. Solutions that integrate relevant T2DM patient data from different sources and which assist the physician in therapeutic decision making, possibly with embedded guideline information, are emerging but not broadly disseminated [21, 22].

In summary, many T2DM patients do not achieve glycemic control and are treated by physicians that are busy handling a breadth of conditions with high administrative burden – leaving little time per patient. At the same time, refined guidelines and a growing number of treatment options add complexity to the treatment choice. Physicians do not have the electronic tools at hand to manage this complexity without additional workload. These factors may all contribute to low guideline adherence.

Therefore, this paper aims to capture the physician perception of guideline adherence and identify leverage points for increasing adherence.

This paper draws on previous research where perspective on guideline adherence was collected from 9 outpatient physicians in Southern Germany and correlated with data from their patients [23]. The present paper aims to augment and validate these initial findings by including feedback of additional diabetes-experienced physicians from different regional and clinical set-ups.

## 2 Subjects, materials, and methods

This study is based on a survey among physicians in primary and specialist practice as well as hospital-based physicians with experience in the treatment of T2DM patients in Germany. Survey participants were recruited in 2 ways: First, customers of Roche Diabetes Care in the Baden-Württemberg region (“BW physicians”) who were asked to complete the questionnaire during routine visits of the sales team. Second, participants of the 2019 Congress of the German Diabetes Association (DDG Diabeteskongress, “DDG physicians”) who were asked to complete the questionnaire during breaks between scientific presentations.

Survey responses were collected with a paper-based questionnaire. The questionnaire was developed by the authors and included:

– 4 demographic questions about physicians' role, experience with T2DM, type of practice, and participation status in the German national T2DM program.
– 1 question about awareness of German national diabetes guidelines
– 2 questions about the perceived value of 4 aspects of the NVL. Physicians were asked to rate each aspect by the difficulty of implementation in daily clinical practice and by the impact on treatment success. The 4 aspects were monitoring adherence, screening adherence, targeting adherence, and therapy adherence Table 1 provides more detailed information about these 4 aspects.

**Table 1:** Physician Survey: 4 NVL Aspects used to assess Difficulty of Implementation and Impact on Treatment Success (excerpt from survey questionnaire).

Monitoring Adherence	At least once per year documentation of medical history, laboratory values, physical and technical examinations (includes weight, blood pressure, peripheral arteries, eye and foot examinations, examinations of the peripheral nervous system, examination of the injection sites in insulin-treated people with diabetes).
Screening Adherence	Annual screening for secondary and concomitant diseases of T2DM: diabetic neuropathy, foot lesions, nephropathy, retinal complications, macrovascular and microvascular risk assessment, an examination of a depressive disorder.
Targeting Adherence	Agreement of individual treatment targets with the patient for lifestyle, glycemic control (blood glucose, HbA1c), LDL cholesterol, weight, and blood pressure.
Therapy Adherence	Adherence to the treatment algorithm “Grundzuege der Behandlung des Typ-2-Diabetes” (basic principles of treatment for type 2 diabetes): If the individual HbA1C treatment target is not reached, diabetes therapy is escalated every 3–6 months from stage 1 (basic therapy) to stage 4 (intensified insulin and combination therapy).
– 2 questions about 13 potential barriers and 9 potential enablers for guideline adherence (Tables 2 and 3).

**Table 2:** Physician survey: Potential barriers to guideline adherence (13 items, [Abbreviation]).

Potential Barriers (B) for Guideline Adherence	
B1	The patient is not able to adequately manage diabetes (e.g. lack of education, cognitive deficiencies, psychosocial problems) [**Patient Inability**].
B2	The patient is not ready or motivated to adequately manage diabetes (especially for lifestyle aspects like diet or exercise) **[Patient Demotivation].**
B3	The patient does not consent to guideline adherent treatment (e.g. antipathy against insulin, fear of side effects) **[Patient Refusal].**
B4	The physician does not have all relevant clinical information (e.g. most recent lab panel, medication) at the time of therapy decision **[Missing Clinical Information].**
B5	The organization of the healthcare system is not well suited for T2DM care **[Health Care System Deficiencies].**
B6	Guideline adherent therapy is not possible for medical reasons (e.g. contraindication) **[Medical Reasons].**
B7	Patients and physicians do not cooperate well in therapy (e.g. missed appointments) **[Patient-Physician Relationship].**
B8	The aims of the guideline are not aligned with the structure of the reimbursement system. **[Nonalignment of Guideline and Reimbursement].**
B9	The physician is not sufficiently informed about the guideline or trained in its use **[Deficient Physician Training].**
B10	The physician disagrees with the guideline recommendation (on certain points). **[Physician Disapproval with Guideline]**
B11	Due to high workload, the physician cannot devote enough time to the individual patient **[High Physician Workload]**
B12	There is a lack of effective coordination between care providers (e.g. outpatient/inpatient, GP/specialist) **[Deficient cross-sectional Coordination].**
B13	The physician lacks the necessary self-confidence to initiate complex therapy regimes - especially pharmaco- and insulin therapy **[Deficient therapeutic Self-Confidence]**

**Table 3:** Physician survey: Potential enablers for guideline adherence.

Potential Enablers (E) for Guideline Adherence	
E1	Reminder function for monitoring and treatment appointments for the practice team or the patient. [**Appointment Reminder Function**]
E2	Better education and training opportunities for patients **[Patient Education]**
E3	Better training opportunities for physicians (e.g. guideline revisions, complex treatment regimens) **[Physician Education]**
E4	Alignment of the reimbursement system with the guideline (e.g. compensation of treatment success and guideline adherence) **[Alignment of Guideline and Reimbursement]**
E5	Financial incentives for patients to reward guideline adherence. **[Patient financial Incentives]**
E6	Better networking and alignment between the parties involved in care (e.g. access to specialists, information exchange) **[Cross-Provider Collaboration]**
E7	Better electronic support of physician in therapy decisions (e.g. important clinical information at a glance, highlighting abnormalities) **[Electronic Therapy Decision Support]**
E8	Better ability for the physician to immediately detect deviations from the guideline (e.g., time intervals for follow-up, therapy algorithm) **[Real-time Guideline Deviation Tracking]**
E9	Revision of the guideline to reflect new research results. **[Guideline Revision]**

The selection of barriers and enablers were informed by previous research [1, 2, 6, 12, 24, 25, 26, 27]. The queried enablers included aspects primarily addressed by digital solutions (Table 3, items 1, 7, 8) which were not the focus of previous research.

Demographic characteristics and guideline awareness were analyzed by calculating absolute and relative frequencies. To assess the perceived value of the NVL, participants were asked to rank the 4 NVL areas (monitoring, screening, targeting, and therapy adherence) by difficulty of implementation and impact on treatment success. For the difficulty of implementation, rank 1 was “easiest to implement” and rank 4 was “most difficult to implement”. For impact on treatment success, rank 1 was “highest influence on treatment success” and rank 4 was “lowest influence on treatment success”. Results were analyzed by combining the 4 ranks to 2 groups (“higher”, “lower”): A response was interpreted as “higher difficulty of implementation” if it was ranked 3 or 4 and as “lower difficulty of implementation” if it was ranked 1 or 2. A response was interpreted as a “higher impact on treatment success” if it was ranked 1 or 2 and interpreted as a “lower impact on treatment success” if it was ranked 3 or 4. These grouped results were analyzed for each NVL area separately and combined (difficulty-impact matrix) by calculating absolute and relative frequencies.

Barriers and enablers for guideline adherence were assessed with a 4-point Likert scale. For barriers, the scale ranged from “1 = never cause for a deviation from the guideline (lower influence)” to “4 = very frequent cause for a deviation from the guideline (high influence)”. For enablers, the scale ranged from “1 = does not increase guideline adherence (low influence)” to “4 = strongly increases guideline adherence (high influence)”. For analysis, the 4 points of the scale were assigned either to the group of “higher” or “lower” influence: Higher influence was assigned to a response when the physician rated a barrier or enabler 3 or 4. Conversely, a lower influence was assigned when the physician rated a barrier or enabler 1 or 2. The grouped results were analyzed for each barrier and enabler by calculating absolute and relative frequencies.

The consistency of results between subgroups was evaluated using Fisher's exact test (FET, α ≤ 0.05 ). 5 different groupings were considered: Physician type (GP vs. Specialist), physician role (owner/department head vs. assistant/salaried physician), physicians' self-reported knowledge of the T2DM treatment guideline (good or very good vs. lower), physician experience with T2DM (>10 years vs. less), and recruitment (BW physicians, DDG physicians).

## 3 Results

118 physicians were invited to participate in the study. 52 physicians filled out the questionnaire (response rate: 44%). 6 questionnaires were excluded from the analysis because 2 participants did not complete the full survey and 4 participants delegated filling out the questionnaires to non-physicians. Table 4 shows the characteristics of the remaining 46 participants included in the analysis.

**Table 4:** Physician characteristics (N = 46).

	n	%
**Physician Type**		
General Practitioner	9	20%
Specialist	37	61%
Outpatient	30	6%
Inpatient	7	13%
**Physician Role**		
Owner/Department Head	36	78%
Assistant/Salaried Physician	10	22%
**NVL Knowledge**		
Good or very good knowledge	42	93%
Partial or no knowledge	3	7%
**T2DM Experience**		
≤10 years	8	17%
>10 years	38	83%
**Recruitment**		
BW Physicians	25	54%
DDG Physician	21	46%
**Participation in German T2DM DMP**	41	89%

Most participants were outpatient diabetology specialists and practice owners and had more than 10 years of experience in treating T2DM. >90% of physicians reported good or very good knowledge of the NVL and participated in the DMP. All physicians who did not participate in the DMP where hospital-based physicians where the DMP does not apply.

**Figure 1:**
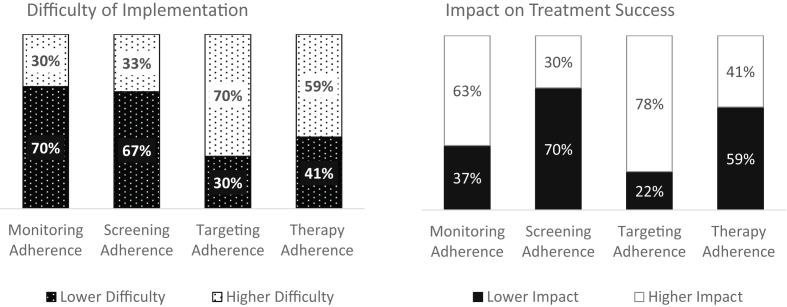
Ranking for Difficulty of Implementation and Impact on Treatment Success in 4 NVL Areas (N = 46, share of responses in %).

Figure 1 shows how physicians ranked difficulty of implementation and impact on treatment success for each of the 4 NVL adherence areas. The responses show a strong consensus among physicians: In all 4 areas and both dimensions (difficulty, impact), there were 59–70% matching answers. The lowest consensus was observed in therapy adherence. Monitoring and screening adherence were considered easier to implement. Monitoring and targeting adherence were considered more impactful for treatment success. Screening adherence was considered the least impactful.

Table 5 shows the ranking of NVL areas by subgroup. For the difficulty of implementation, there were no significant differences between the 5 subgroups analyzed. For impact on treatment success, the comparison of subgroups identified significant differences in 2 areas: Owners/department heads (owners) considered screening adherence less impactful than salaried/assistant physicians (assistants, p = 0.047). BW physicians considered monitoring adherence more impactful than DDG physicians did (p = 0.002). The data allowed us to observe a trend of large differences between subgroups in the assessment of how impactful monitoring adherence is for treatment success. The share of physicians who attest to a higher impact differs by ≥ 30 percentage points in 4 of 5 subgroups.

**Table 5:** Ranking for Difficulty of Implementation and Impact on Treatment Success: (n = 46): Share of participants who ranked each of the 4 NVL areas higher difficulty/higher impact, by Subgroups.

	Total	Physician Type			Physician Role			NVL Knowledge			T2DM Experience			Recruitment		
		GP	Specialist	*FET* *p*	Owner	Assistant	*FET* *p*	Lower	higher	*FET* *p*	≤10 years	>10 years	*FET* *p*	BW Physicians	DDG Physicians	*FET* *p*
N	46	9	37		36	10		3	42		8	38		25	21	

Higher Difficulty of Implementation																
Monitoring	30%	33%	30%	*1.000*	31%	30%	*1.000*	33%	31%	*1.000*	50%	26%	*0.220*	24%	38%	*0.349*
Screening	33%	33%	32%	*1.000*	42%	0%	*0.190*	0%	36%	*0.540*	13%	37%	*0.243*	36%	29%	*0.754*
Targeting	70%	67%	70%	*1.000*	69%	70%	*1.000*	100%	69%	*0.546*	63%	71%	*0.684*	68%	71%	*1.000*
Therapy	59%	44%	62%	*0.456*	56%	70%	*0.488*	33%	62%	*0.555*	63%	58%	*1.000*	56%	62%	*0.769*

Higher Impact on Treatment Success																
Monitoring	63%	89%	57%	*0.124*	64%	60%	*1.000*	100%	60%	*0.279*	88%	58%	*0.226*	**84%**	**38%**	***0.002***
Screening	30%	33%	30%	*1.000*	**22%**	**60%**	***0.047***	67%	26%	*0.196*	13%	34%	*0.403*	24%	38%	*0.349*
Targeting	78%	78%	78%	*1.000*	81%	70%	*0.666*	33%	81%	*0.119*	75%	79%	*1.000*	76%	81%	*0.735*
Therapy	41%	44%	41%	*1.000*	39%	50%	*0.719*	67%	38%	*0.555*	50%	39%	*0.700*	40%	43%	*1.000*

FET = Fisher's Exact Test, significant results in **bold**. The significance threshold for the p-values in italics is α ≤ 0.05.

**Figure 2:**
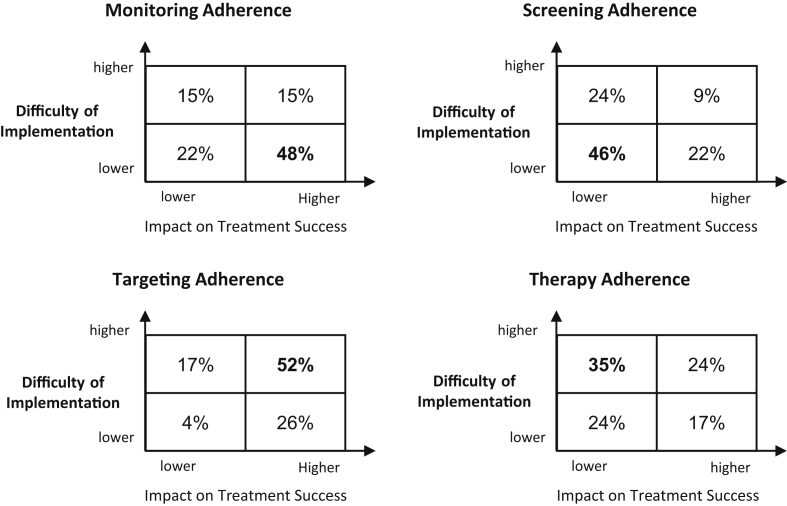
Difficulty-Impact Matrix in 4 NVL Areas (N = 46, share of responses in %).

Figure 2 combines the ranking results for the difficulty of implementation and impact on treatment success in a difficulty-impact matrix. The matrix shows that the dominant quadrant with 35–52% of responses is different for each 4 NVL area. Strongest consensus between physician responses was achieved in targeting adherence: 52% of participants rate the area as difficult to implement and impactful on treatment success.

Table 6 shows the results of the participants' rating of 13 potential barriers to guideline adherence. Patient inability (B1) and patient demotivation (B2) were considered the highest barriers for guideline adherence. Other barriers that scored high were a dysfunctional patient-physician relationship (B7), high physician workload (B11), and deficient coordination between health care sectors (B12). Items that physicians scored as lower barriers were the organization of the health care system (B5), medical reasons (B6), and a lack of physician self-confidence to initiate complex therapy regimes (B13).

**Table 6:** Physician Rating of Potential Barriers for Guideline Adherence by Subgroups (N = 46): Share of responses rating the item as very often or frequently a reason for non-adherence.

Items very often or frequently rated as Reason for Non-adherence	Total	Physician Type			Physician Role			NVL Knowledge			T2DM Experience			Recruitment		
		GP	Specialist	*FET* *p*	Owner	Assistant	*FET* *p*	Lower	higher	*FET* *p*	≤10 years	>10 years	*FET* *p*	BW Physicians	DDG Physicians	*FET′* *p*
N	46	9	37		36	10		3	42		8	38		25	21	

B1 Patient Inability	93%	100%	92%	*1.000*	92%	100%	*1.000*	100%	93%	*1.000*	100%	92%	*1.000*	96%	90%	*0.585*
B2 Patient Demotivation	78%	89%	76%	*0.659*	75%	90%	*0.420*	100%	76%	*1.000*	75%	79%	*1.000*	84%	71%	*0.475*
B3 Patient Refusal	54%	67%	51%	*0.478*	58%	40%	*0.475*	67%	52%	*1.000*	63%	53%	*0.710*	64%	43%	*0.235*
B4 Missing Clinical Information	41%	44%	41%	*1.000*	39%	50%	*0.719*	33%	43%	*1.000*	63%	37%	*0.246*	36%	48%	*0.550*
B5 Health Care System Deficiencies	30%	22%	32%	*0.701*	25%	50%	*0.242*	33%	31%	*1.000*	38%	29%	*0.684*	24%	38%	*0.349*
B6 Medical Reasons	35%	44%	32%	*0.698*	33%	40%	*0.720*	67%	31%	*0.254*	38%	34%	*1.000*	40%	29%	*0.538*
B7 Patient-Physician Relationship	65%	78%	62%	*0.463*	69%	50%	*0.283*	67%	64%	*1.000*	75%	63%	*0.694*	64%	67%	*1.000*
B8 Non-alignment of Guideline and Reimbursement	54%	67%	51%	*0.478*	50%	70%	*0.306*	67%	55%	*1.000*	63%	53%	*0.710*	60%	48%	*0.553*
B9 Deficient Physician Training	50%	56%	49%	*1.000*	50%	50%	*1.000*	33%	52%	*0.608*	50%	50%	*1.000*	44%	57%	*0.554*
B10 Physician Disapproval of Guideline	41%	33%	43%	*0.716*	47%	20%	*0.160*	33%	40%	*1.000*	**0%**	**50%**	***0.014***	48%	33%	*0.377*
B11 High Physician Workload	67%	67%	68%	*1.000*	61%	90%	*0.132*	67%	67%	*1.000*	75%	66%	*1.000*	76%	57%	*0.216*
B12 Deficient cross-sectoral Coordination	63%	78%	59%	*0.450*	61%	70%	*0.723*	100%	60%	*0.279*	75%	61%	*0.691*	72%	52%	*0.225*
B13 Deficient therapeutic Self-Confidence	37%	33%	38%	*1.000*	33%	50%	*0.462*	67%	36%	*0.547*	38%	37%	*1.000*	32%	43%	*0.545*

FET = Fisher's Exact Test, significant results in **bold**. The significance threshold for the p-values in italics is α ≤ 0.05.

Subgroup analysis showed no significant differences with regards to adherence barriers, except for item 10: None of the physicians with ≤10 years of experience vs. 50% of physicians with >10 years of experience in treating T2DM patients viewed disapproval with the guideline as a reason for guideline non-adherence (p = 0.014).

Table 7 shows the participants' rating of 9 potential enablers for increasing guideline adherence. The 3 strongest enablers were better education and training opportunities for patients (B2), better networking and alignment between the parties involved in care (B6), and better electronic support of physicians in therapy decisions (B7). 78–85% of physicians stated that these items could improve adherence. Participants were divided on the topic of paying patients for good adherence (B5): 50% stated that these incentives would improve adherence, and 50% disagreed.

**Table 7:** Physician Rating of Potential Enablers for Guideline Adherence by Subgroups (N = 46): Share of responses rating the item as very often or frequently improving adherence.

Items very often or frequently rated as Enabler for Adherence	Total	Physician Type			Physician Role			NVL Knowledge			T2DM Experience			Recruitment		
		GP	Specialist	*FET* *p*	Owner	Assistant	*FET* *p*	Lower	higher	*FET* *p*	≤10 years	>10 years	*FET* *p*	BW Physicians	DDG Physicians	*FET* *p*
N	46	9	37		36	10		3	42		8	38		25	21	

E1 Appointment Reminder System	65%	78%	62%	*0,463*	67%	60%	*0.720*	33%	67%	*0.285*	63%	66%	*1.000*	64%	67%	*1.000*
E2 Patient Education	78%	78%	78%	*1.000*	72%	100%	*0.089*	67%	79%	*0.539*	88%	76%	*0.664*	84%	71%	*0.475*
E3 Physician Education	67%	89%	62%	*0.235*	64%	80%	*0.460*	33%	69%	*0.254*	88%	63%	*0.243*	56%	81%	*0.115*
E4 Alignment of Guideline and Reimbursement	70%	78%	68%	*0.701*	**61%**	**100%**	***0.020***	67%	69%	*1.000*	75%	68%	*1.000*	64%	76%	*0.522*
E5 Patient Financial Incentives	50%	67%	46%	*0.459*	50%	50%	*1.000*	67%	50%	*1.000*	**88%**	**42%**	***0.047***	56%	43%	*0.554*
E6 Cross-provider Collaboration	85%	89%	84%	*1.000*	83%	90%	*1.000*	100%	83%	*1.000*	100%	82%	*0.325*	84%	86%	*1.000*
E7 Electronic Therapy Decision Support	80%	78%	81%	*1.000*	78%	90%	*0.659*	67%	81%	*0.497*	88%	79%	*1.000*	88%	71%	*0.264*
E8 Real-time Guideline Deviation Tracking	59%	44%	62%	*0.456*	56%	70%	*0.488*	33%	60%	*0.565*	75%	55%	*0.440*	64%	52%	*0.550*
E9 Guideline Revision	70%	78%	68%	*0.701*	75%	50%	*0.242*	67%	69%	*1.000*	63%	71%	*0.684*	72%	67%	*0.755*

FET = Fisher's Exact Test, significant results in **bold**. The significance threshold for the p-values in italics is α ≤ 0.05.

Subgroup analysis for adherence enablers showed significant differences in 2 areas: All assistant and salaried physicians but only 61% owners and department heads believed that alignment of guideline and reimbursement system would increase adherence (p = 0.020). 88% of physicians with ≤10 years of T2DM experience but only 42% of physicians with >10 years of T2DM experience rated financial incentives for patients as an adherence enabler (p = 0.047). As a trend, the largest differences between subgroups (16–36%) were observed in physicians' education as a possible enabler for adherence.

## 4 Discussion

This study shows that T2DM-experienced physicians in Germany have a consistent perspective on the value of the NVL. Participants rated patient-related factors as the strongest barriers for guideline adherence, followed by deficiencies in the patient-physician relationship and high physician workload. Physicians considered electronic physician support systems and improved cross-sectoral collaboration as the strongest enablers for improved guideline adherence.

In many areas, this study aligns with previous research: Studies have shown that physicians often do not intensify T2DM therapy despite knowing that it is indicated (clinical inertia) [6, 27]. This may be related to the findings of this study where physicians consider therapy adherence rather difficult to achieve and having a lower impact on treatment success. Physicians seemed to hesitate to intensify treatment, as recommended by the guideline because they did not believe it would be successful and at the same time a burden for patients [25]. A physician survey by Genere et al. found that targeting adherence is both impactful for treatment success and difficult to implement [28]. 2 European studies observed higher adherence rates for monitoring adherence than for screening adherence parameters [9, 29]. This may indicate that screening is less of a priority because it is perceived to have less of an impact on treatment success. Higher monitoring adherence may indicate that physicians consider monitoring to be both easy to implement and impactful for outcomes.

Barriers and enablers for adequate T2DM care have been studied previously. A review article identified patients' and physicians' beliefs and attitudes as major factors for adherence. Beyond that, physician adherence was also influenced by their diabetes knowledge. And, patient adherence was also influenced by their health literacy, financial situation, comorbidities, and social support system [1]. A more recent review article identified anxiety and uncertainty about how to best manage diabetes as a key barrier among physicians and patients [2]. Barriers to guideline-adherent care were also discussed in the cross-national DAWN2 study. The study describes deficiencies in the health care system and education of physicians as well as inadequate patient self-management capabilities and education as key barriers for adequate diabetes care [30]. A survey among GPs found that patient resistance or refusal and lack of self-management skills were key barriers for initiating insulin therapy [26]. The study states that a strong emphasis on patient factors is common among physicians, similar to the results of this study.

DAWN2 also researched enablers for T2DM guideline adherence. Health providers in 13 countries rated patient education, provider education, and availability of additional specialized resources, and better collaboration within the care team as key areas for improvement in diabetes care [30]. Lower scores were given to financial incentives. These results are largely in line with research by Ross et al., Zafar et al. and the findings of this study [12, 27] Recommendations for overcoming adherence problems include providing physicians with adequate time, infrastructure, resources, and training and incentivize them to provide guideline-adherent care [1]. In addition to these more technical and rationalist aspects, it is important to address patients' and physicians' emotional barriers, e.g. by training physicians on how to achieve behavioral changes among their patients [2]. The participants of this study consider patient-related factors as greater adherence barriers than physician-related factors but assigned high scores to physician-related enablers. This suggests that physicians see the potential to improve the care process they lead. In this study, physicians considered improved cross-provider collaboration to be the highest-rated enabler. This need for integrating care is also reflected in a recent consensus statement by ADA and EASD advocating for an integrated, patient-centered approach to T2DM care [20]. The ongoing digital transformation in healthcare can support this integration with longitudinal care documentation and information exchange between participants in the care process.

Electronic therapy decision support for physicians was the enabler which scored second highest in this study. When guideline information is embedded in these systems, when they can track whether monitoring and screening tasks are completed in adequate intervals, highlight contraindications, and alert about thresholds for therapy intensification, these systems can support physicians in clinical decision making. In recent years, information technology interventions, including clinical decision support systems (CDSS), have shown to improve glycemic control in people with T2DM. Some studies also report improvements in the care process and patient satisfaction [31]. Previous studies had reported some evidence for a positive impact of CDSS on outcomes but claimed that studies had been of low quality and that these findings required further confirmation [32]. CDSS improves practitioner performance, especially when they prompt users automatically instead of requiring users to initiate the system [33].

This study had strengths and limitations. The forced ranking of NVL areas for the difficulty to implement and the impact on treatment success methodologically only allows analysis of relative differences, not the absolute perception of “high” or “low”. This may have led to over- or understating physicians' responses. This study is based on self-reporting. Results reflect the subjective perception of physicians. Selection bias may have occurred in the group of physicians who were invited to participate by Roche Diabetes Care. However, there was very little difference in how these physicians rated compared to the randomly selected participant group recruited at the DDG congress. The sample size in some of the subgroups is very small and p-values must be interpreted with caution. A larger sample size in these subgroups would be required to confirm consistency between groups. As a strength, this study was able to capture feedback on a broad set of adherence barriers and enablers from the same group of respondents, which allowed an interesting perspective on physician-related factors influencing guideline adherence. For the first time, the role of data-driven, digital aspects are researched in this context. Our study showed the value of different, typical aspects of T2DM treatment guidelines to physicians. It is, to our knowledge, the first assessment of physician perspective on T2DM guideline adherence in the German-speaking area.

## 5 Conclusion

This study suggests that physicians consider patient factors to be stronger adherence barriers than physician factors. Opportunities to increase physician adherence must be leveraged in addition to increasing patient competence and engagement and building a health care system suitable for chronic care. Physicians voiced appreciation for adherence enablers based on digital solutions. With the high physician workload and often challenging patient-physician relationship, such enablers must be designed so that they do not increase workload and complexity for physicians. Existing practice management systems could be enhanced with automated appointment reminders and follow-up functionalities, with patient dashboards integrating key data from different sources, or with the ability to immediately see deviations from most current guidelines. This can save valuable physician time, increase evidence-based decision making, and allow physicians to refocus on the conversation with their patients.

## Declarations

### Author contribution statement

S. Brenner: Conceived and designed the experiments; Performed the experiments; Analyzed and interpreted the data; Contributed reagents, materials, analysis tools or data; Wrote the paper.

W. Oberaigner: Conceived and designed the experiments; Analyzed and interpreted the data; Wrote the paper.

H. Stummer: Analyzed and interpreted the data; Contributed reagents, materials, analysis tools or data; Wrote the paper.

### Funding statement

This research did not receive any specific grant from funding agencies in the public, commercial, or not-for-profit sectors.

### Competing interest statement

The authors declare no conflict of interest.

### Additional information

No additional information is available for this paper.
